# GTV delineation in supraglottic laryngeal carcinoma: interobserver agreement of CT versus CT-MR delineation

**DOI:** 10.1186/s13014-014-0321-4

**Published:** 2015-01-23

**Authors:** Elise Anne Jager, Nicolien Kasperts, Joana Caldas-Magalhaes, Mariëlle EP Philippens, Frank A Pameijer, Chris HJ Terhaard, Cornelis PJ Raaijmakers

**Affiliations:** Department of Radiation Oncology, University Medical Center Utrecht, Q01.118; Heidelberglaan 100, 3584 CX Utrecht, the Netherlands

**Keywords:** Interobserver agreement, Supraglottic laryngeal carcinoma, Head-and-neck cancer, GTV, MRI

## Abstract

**Background:**

GTV delineation is the first crucial step in radiotherapy and requires high accuracy, especially with the growing use of highly conformal and adaptive radiotherapy techniques. If GTV delineations of observers concord, they are considered to be of high accuracy.

The aim of the study is to determine the interobserver agreement for GTV delineations of supraglottic laryngeal carcinoma on CT and on CT combined with MR-images and to determine the effect of adding MR images to CT-based delineation on the delineated volume and the interobserver agreement.

**Methods:**

Twenty patients with biopsy proven T1-T4 supraglottic laryngeal cancer, treated with curative intent were included. For all patients a contrast enhanced planning CT and a 1.5-T MRI with gadolinium were acquired in the same head-and-shoulder mask for fixation as used during treatment. For MRI, a two element surface coil was used as a receiver coil. Three dedicated observers independently delineated the GTV on CT. After an interval of 2 weeks, a set of co-registered CT and MR-images was provided to delineate the GTV on CT. Common volumes (C) and encompassing volumes (E) were calculated and C/E ratios were determined for each pair of observers. The conformity index general (CIgen) was used to quantify the interobserver agreement. Results: In general, a large variation in interobserver agreement was found for CT (range: 0.29-0.77) as well as for CT-MR delineations (range: 0.17-0.80). The mean CIgen for CT (0.61) was larger compared to CT-MR (0.57) (p = 0.032). Mean GTV volume delineated on CT-MR (6.6 cm^3^) was larger compared to CT (5.6 cm^3^) (p = 0.002).

**Conclusion:**

Delineation on CT with co-registered MR-images resulted in a larger mean GTV volume and in a decrease in interobserver agreement compared to CT only delineation for supraglottic laryngeal carcinoma.

**Electronic supplementary material:**

The online version of this article (doi:10.1186/s13014-014-0321-4) contains supplementary material, which is available to authorized users.

## Background

Radiotherapy for head-and-neck cancer can give rise to severe acute and late side effects [1-4]. To minimize damage to healthy tissues on one hand and eradicate macroscopic tumor on the other hand, the gross tumor volume (GTV) should be determined as accurate as possible. This is especially required when applying intensity-modulated radiation therapy (IMRT) and position verification, to maximize the benefits of high-precision radiation techniques using smaller radiation fields [5].

Various studies [6-10] have been performed, using different imaging modalities, to determine the agreement among observers when delineating the GTV in head-and-neck cancer. Interobserver agreement and interobserver variability (disagreement) are often used in the same context whereas these terms express the opposite. Generally, interobserver agreement is used to assess the quality of an image modality to visualize the tumor. Thus, when there is more agreement among the observers, the image modality is assumed to be more precise and even more accurate in visualizing the tumor, although high accuracy can only be assessed by pathology.

For delineating the GTV and treatment planning in head-and-neck cancer, Computed Tomography (CT) is the imaging modality of first choice in most cases [11,12]. The advantages of CT are that it is widely available, does not cause geometrical distortion and has intrinsic information on the relative electronical density of the various tissues used for dose calculation algorithms [11]. Where CT offers excellent bony detail, magnetic resonance imaging (MRI) uses various sequences to visualize soft tissue and bone contrasts. Especially the capability of MRI to visualize soft tissues is an improvement compared to CT, therefore permitting better definition of disease extent and organs at risk [12-14]. Because MRI does not carry intrinsic information on electronic density, it is currently precluded as sole imaging modality in clinical use for radiotherapy treatment planning in head-and-neck tumors [11,12]. Various studies demonstrated superior soft tissue contrast on MRI compared to CT [6,9,15,16]. Although there is agreement on the capacity of MRI to increase visibility of soft tissue structures in head-and-neck oncology, there is no agreement on the value of MRI for determination of the GTV and its influence on the interobserver agreement [7,8,10,11].

The aim of this study is to compare the interobserver agreement between delineations on CT and on CT with co-registered MR-images in supraglottic laryngeal carcinoma and to determine the value of adding MR-images to the “gold standard” CT images.

## Methods

### Patient selection

Twenty patients with biopsy proven T1-T4 supraglottic laryngeal cancer (squamous cell carcinoma, SCC) and treated with high-dose radiotherapy with curative intent at our institution between November 2005 and October 2009 were included in this study.

From a database of 120 patients with laryngeal and hypopharyngeal cancer, 39 patients fulfilled the criteria of inclusion. Which were; patients with a supraglottic tumor, the availability of a contrast enhanced CT scan and a MRI with gadolinium performed in a radiotherapy mask. Twenty patients were randomly selected from this group, Initial clinical assessment of tumor stage was performed based on triple-endoscopy under anesthesia, contrast enhanced CT-scan, and indirect laryngoscopy to assess mobility of the vocal cords. The study group consisted of five female and 15 male patients with a mean age of 64 years (range: 40-80 yr).

### Imaging technique and data acquisition

**Figure 1:**
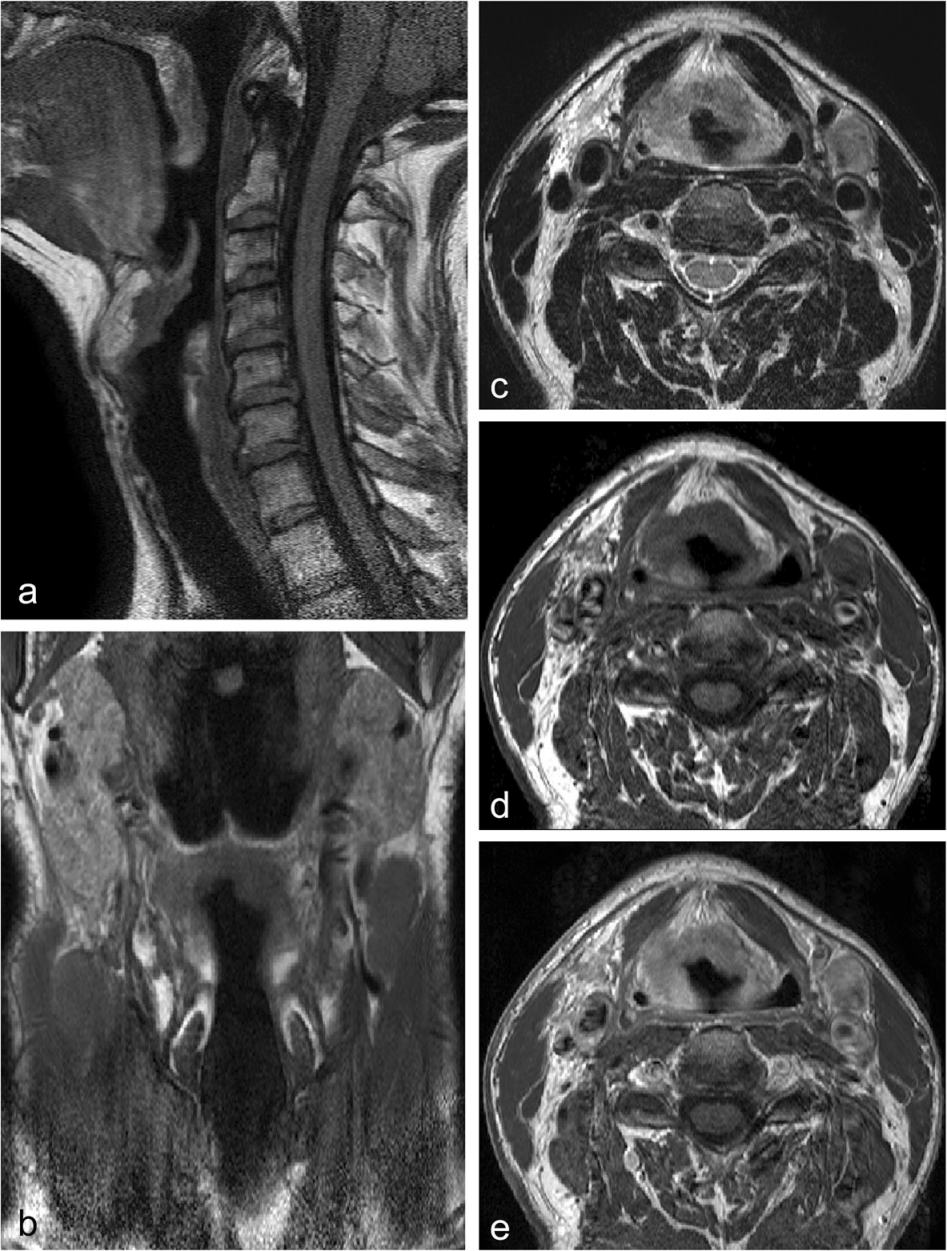
**MR-images of the larynx acquired in a radiotherapy mask. a**:T1-weighted sagittal view, **b**: T1-weighted coronal view. Transversal views: **c**: T2-weighted, **d**: T1-weighted, **e**: T1-weighted + gadolinium.

CT and MR-imaging was performed prior to radiotherapy treatment and in radiotherapy position. Patients were immobilized in a radiotherapy mask (five-point head-and-shoulder mask, Posicast PR5; Civco, Reeuwijk, The Netherlands) and received a CT-scan from the base of the skull to the carina after intravenous injection of iodinated contrast. The CT-images were obtained by two different CT-machines. Fifteen patients were scanned with a single slice Philips Aura machine and five patients with a Philips Big Bore Brilliance (multi-slice CT). Images were acquired with helical scans. A slice thickness of 2 mm and 3 mm, and a pitch of 1.0 (Philips Aura) and 0.7 (Philips Big Bore Brilliance) was used. Axial images were acquired using a matrix size of 512 × 512, with a pixel spacing of 0.95 × 0.95 mm^2^ - 1.19 × 1.19 mm^2^. After a mean interval of six days (range: 0-13 days) the patients underwent a 1.5 Tesla MRI-scan (Achieva; Philips Medical System, Best, the Netherlands) in the same fixation device as used for CT-scanning, combined with a two-element flexible surface coil [17,18]. For every patient T1-weighted images were obtained in transversal, sagittal and coronal directions as well as transversal T2-weighted and T1-weighted after injection of gadolinium according to our clinically used MR protocol for imaging the larynx and the hypopharynx. A 512 acquisition and reconstruction matrix was used. The field-of-view diameter was 210 mm and the slice thickness was 3 mm. An example of the acquired MR-images is shown in Figure 1. The registration was performed by a medical physicist who defined a rectangular box containing the GTV and surrounding bony structures. The rigid registration was performed using the mutual information algorithm within this box and the registration was visually controlled. This procedure is according to clinical practice at our department. No approval of an ethics committee was needed according to Dutch law.

### Delineation of GTV

Gross tumor volume (GTV) was defined as the macroscopic (gross) extent of the primary tumor that is demonstrable on the imaging modality e.g, MRI-scan, CT-scan. The following guidelines for delineation of the GTV were agreed upon by the three observers at a consensus meeting in advance of the delineations sessions. Areas of doubt had to be included in the GTV according to radiotherapy practice. Edema around the tumor had to be included and evident stasis of saliva had to be excluded in the delineation. Criteria for soft tissue infiltration were: left-right asymmetry, contrast enhancement and fatty space infiltration. For cartilage invasion on CT the following signs were used: osteolysis of dense mineralized areas (if in contact with the primary tumor), cortical erosions, abnormal increased asymmetrical density and presence of tumor on both sides of bony/cartilaginous structures. Sclerotic cartilage with an intact cortex was not to be included in the GTV. Guidelines for interpretation of neoplastic invasion of laryngeal cartilages, as defined by M. Becker et al. [19], were used during delineation on MRI.

Three dedicated and MRI-trained head-and-neck specialists (two radiation oncologists and one radiologist) respectively called observer a, b and c, independently delineated the GTV. They started with CT-images at fixed window/level 350/50 with minor adjustments of 10-20 HU based on individual preferences.

After an interval of more than two weeks, to avoid possible bias due to recall of the previous delineation, the same CT (without previous contours) was delineated with the co-registered MR-images (T1w, T1w + Gd, T2w) simultaneously visible. Typical examples of delineations on CT and CT-MR can be found in the Additional files 1, 2, 3 and 4.

The observers received an anonymised triple-endoscopy report and were instructed to record: delineation time, window/level and which anatomical parts of the larynx were involved by tumor, during delineating on CT and CT-MR. Observers were also asked to subjectively rate image quality (good, moderate, poor and not assessable) and tumor detectability [20]. The latter was scored as followed; 0, if tumor boundaries were not visible, 1: tumor is visible, boundaries not, 2: boundaries are visible but not clear, 3: tumors as well as boundaries are clearly visible.

### Volumetric analysis and interobserver agreement

All GTVs were delineated in volume tool [21], a software application that is capable of simultaneous visualization of multiple 3-dimensional datasets. The volume of the GTV was determined by multiplying the number of voxels contained within a contour by the size of the voxel. The size of the voxel depends on the resolution of the image reconstruction and the slice thickness. If the center of the voxel is within the contour boundary, the voxel is regarded as being part of the volume.

For each pair of observers, the common volume (C; the volume that is part of both GTVs of one patient) and the encompassing volume (E; volume encompassing both GTVs of one patient) of the delineated GTVs for each patient, were automatically calculated. C/E ratios (Jaccard coefficient) were determined for each pair of observers (observer a&b, a&c, b&c). The Jaccard coefficient can only be used as a conformity index for a situation in which two delineated volumes are compared. Therefore we used the conformity index general (CIgen) to quantify the agreement between all observers. This index is independent of the number of observers or delineated volumes [22]. CIgen is defined as the sum of the common volumes of the various observer pairs divided by the sum of the encompassing volumes of these pairs and is written for the 3 observers as the following formula:

$$ C{I}_{gen}=\frac{\left(a\cap b\right)+\left(a\cap c\right)+\left(b\cap c\right)}{\left(a\cup b\right)+\left(a\cup c\right)+\left(b\cup c\right)} $$

A CIgen of 1.00 indicates perfect overlap (identical delineations), whereas a CIgen of 0.00 indicates no overlap at all.

### Clinical impact

Since the GTV is extended using margins to correct for several factors such as microscopic disease, movement and setup inaccuracies, the planning target volume is considerably larger than the GTV.

For each patient the GTV delineations were extended with a margin to create a PTV. Two scenarios were investigated according to the work of Vugts et al. [23]. In one scenario conventional margins were applied (PTV_clinical_). In the other scenario tight margins were investigated (PTV_tight_). A margin of 8 mm was used for PTV_tight_ and15 mm for PTV_clinical_. As a “worst case scenario”, the largest GTV was assumed to be the correct GTV. Subsequently, it was determined in how many cases this GTV was not covered by the PTVs.

### Statistical analysis

Based on non-normality of the samples according to the Shapiro Wilk test, the Wilcoxon signed-rank test was applied for statistical comparison of the mean delineated volume (GTV) between CT and CT-MR.

The coefficient of variation (COV), defined as COV = standard deviation (SD)/mean volume, was determined for all delineated GTVs of the patients for each imaging modality. For each modality correlation between mean GTV volume and COV was measured using a Spearman rank correlation test.

A Student paired t-test was used for the comparison of the CIgen on CT and CIgen on CT-MR. These samples were both normally distributed according to the Shapiro Wilk test. For each modality the correlation between CIgen and GTV volume was measured using a Spearman rank correlation test. Statistical analyses were performed with SPSS 16.0 using a (alpha) level of significance of 0.05.

## Results

Image quality was considered “good” for the majority of the CT-images as well as for MR-images. For some patients the image quality of the CT-scan was deteriorated by contrast insufficiency or due to swallowing. Movement due to swallowing had an adverse effect on MR-image quality. However, the image quality was never considered to be “not assessable”, nor did the observers unanimously qualify the image quality as being “poor”.

For the CT of 16 patients, at least one observer recorded that the tumor borders were “visible but not clear” or worse (grade 2, 1 and 0). For MRI, this was the case for 11 patients. In eight patients at least one of the observers recorded explicit difficulties in the cranial and/or caudal direction on CT and in four patients referring to the MRI. These recorded difficulties in cranial and caudal direction were objectified by larger discrepancies (smaller common volumes) in the delineations in the cranial and caudal areas and in de region of the epiglottis for CT as well as for MRI in nearly all patients.

### Volumetric analysis

The difference between GTV volume on CT and on CT-MR was considerably larger in two cases (patient 10 and 15) (Table 1) compared to the difference for the remaining patients.

**Table 1:** **Gross tumor volumes delineated by 3 observers on CT and CT-MR in supraglottic laryngeal carcinoma**

**Patient (no.)**	**Tumor stage**	**Mean GTV (cm** ^**3**^ **)**		**CIgen**		**COV**	
		**CT**	**CT-MR**	**CT**	**CT-MR**	**CT**	**CT-MR**
**8**	T1	1.2	1.3	0.52	0.54	0.23	0.11
**6**	T2	1.5	1.9	0.29	0.32	0.41	0.66
**12**	T3	1.6	1.5	0.62	0.67	0.18	0.03
**16**	T3	1.9	2.8	0.43	0.17	0.38	0.13
**20**	T2	2.3	2.8	0.61	0.41	0.14	0.38
**14**	T2	2.9	3.2	0.73	0.68	0.07	0.05
**9**	T2	5.0	5.1	0.65	0.61	0.17	0.25
**4**	T2	5.3	5.2	0.62	0.50	0.19	0.41
**1**	T3	5.5	6.7	0.76	0.66	0.04	0.06
**19**	T2	5.5	6.6	0.63	0.54	0.04	0.06
**5**	T2	5.7	6.7	0.49	0.49	0.32	0.30
**11**	T2	6.8	6.5	0.68	0.69	0.18	0.14
**13**	T2	7.0	6.8	0.77	0.80	0.10	0.03
**7**	T2	7.1	7.3	0.75	0.73	0.04	0.12
**2**	T3	7.9	9.6	0.70	0.59	0.09	0.27
**18**	T3	7.9	8.1	0.59	0.57	0.10	0.08
**17**	T3	11.1	11.8	0.67	0.62	0.05	0.15
**10**	T4	12.8	22.1	0.49	0.50	0.36	0.35
**15**	T4	14.7	20.6	0.69	0.68	0.09	0.16
**3**	T3	27.0	29.1	0.60	0.66	0.26	0.11

Tumor stage, mean delineated tumor volumes (GTV) in cm^3^ of 3 observers ranked by mean GTV. COV (coefficient of variation) and CIgen (conformity index general) for GTV on CT and CT-MR.

The median GTV volume of the three observers on CT-MR (median: 6.6 cm^3^, interquartile range: 2.9-9.2, 95% confidence interval: 4.8-11.8 cm^3^) was significantly larger (p = 0.002) compared to median of the GTV volume on CT (median: 5.6 cm^3^, interquartile range: 2.5-7.9, 95% confidence interval: 4.2-9.8 cm^3^) (Table 1).

A large variation in COV was found between the GTV per modality as well as between the modalities (Table 1). The mean COV on CT (0.17, SD 0.12) was comparable to CT-MR (0.19, SD 0.16) (Table 1).

No relation between COV and the mean GTV volume for any of the imaging modalities was observed (CT: rho = -0.28 p = 0.23 CT-MR: rho *=* 0.05 p = 0.84).

### Interobserver agreement

**Figure 2:**
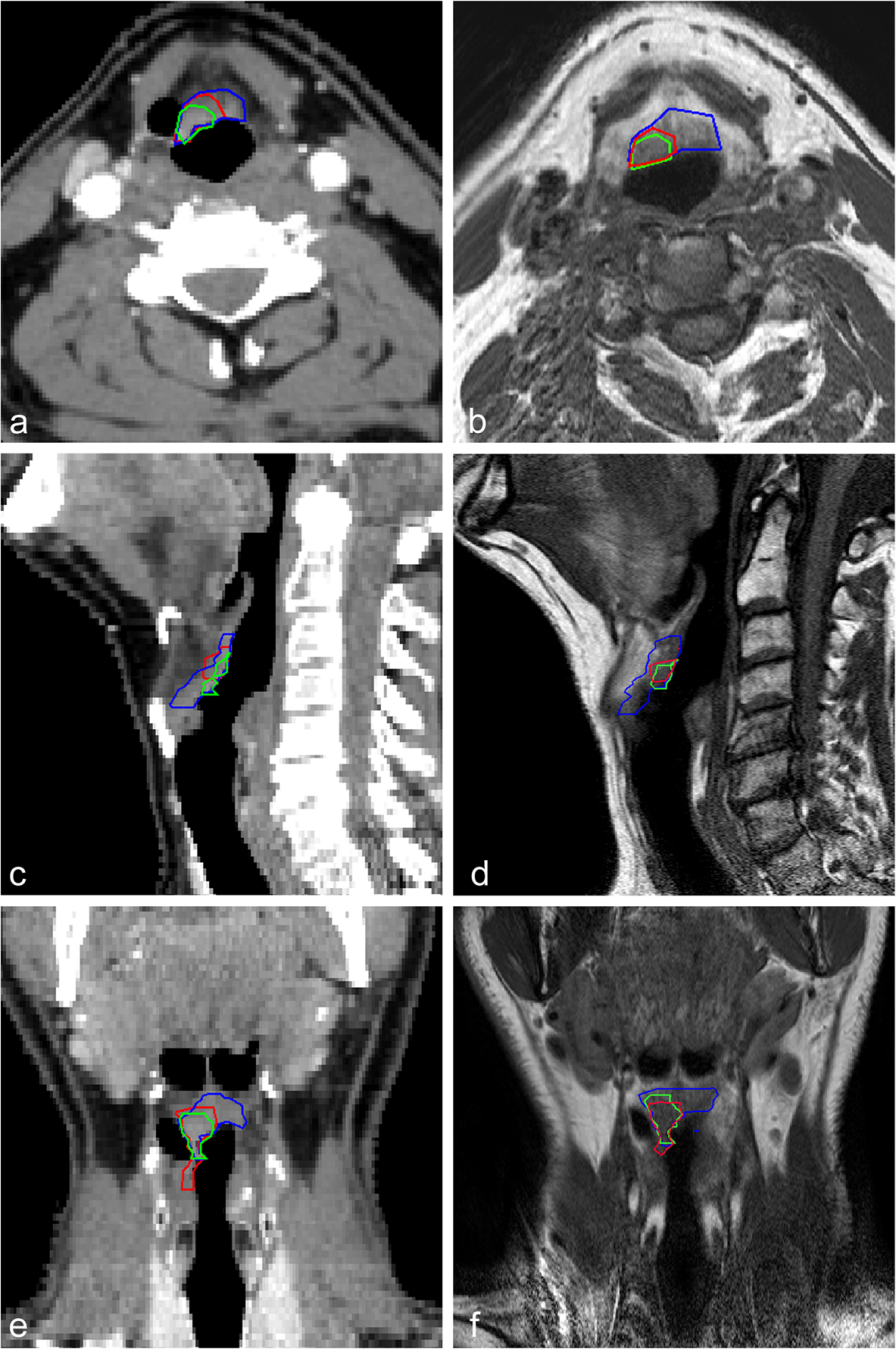
**Delineations of three observers on contrast enhanced CT and CT-MR.** Delineations of three observers on contrast enhanced CT **(a, c, e)** and CT-MR **(b, d, f)** (T1-weighted). Transversal **(a, b)**, sagittal **(c, d)** and coronal **(e, f)** views.

**Figure 3:**
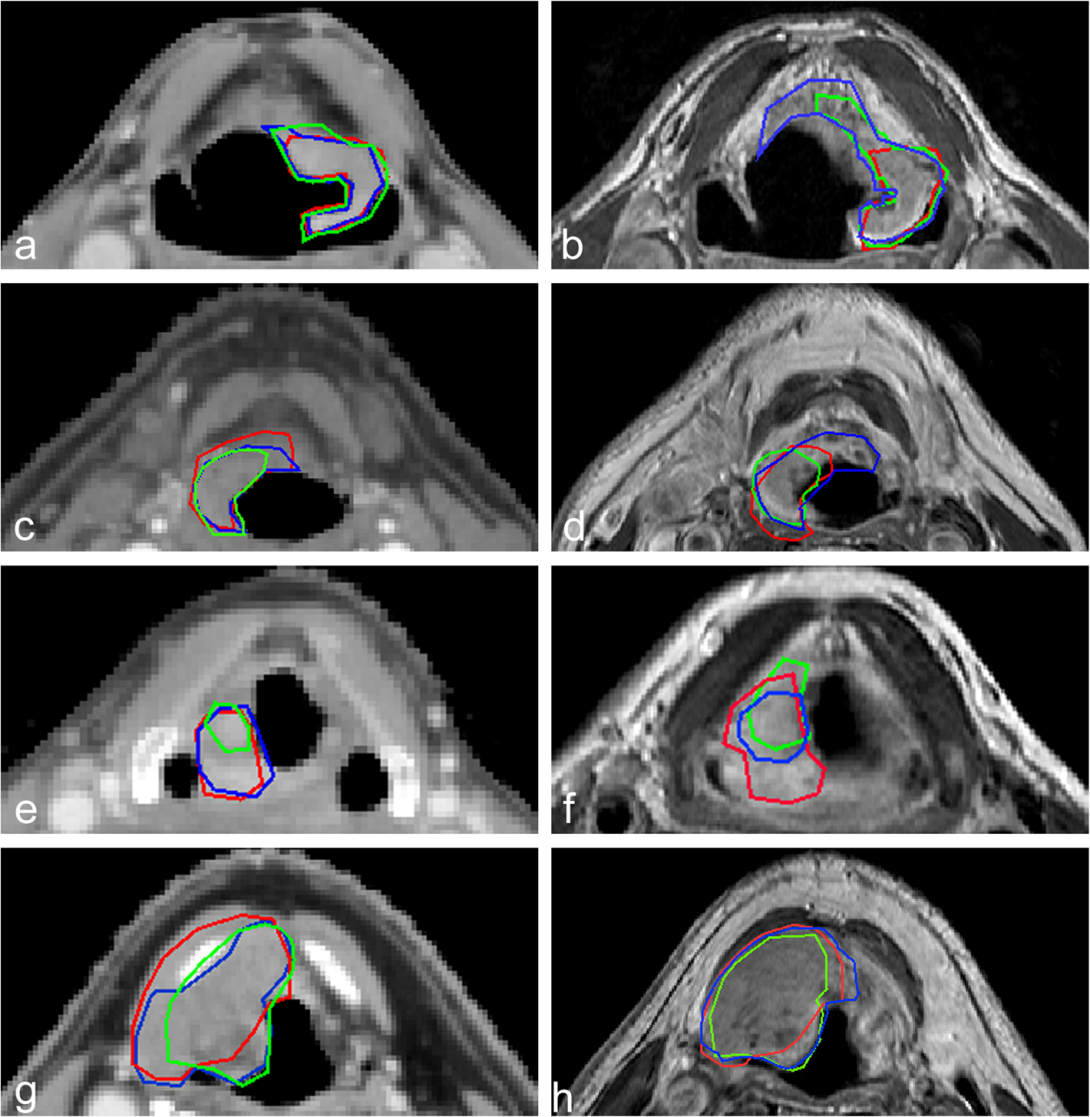
**Delineations of three observers on contrast enhanced CT and CT-MR.** Delineations of three observers on contrast enhanced CT **(a, c, e, g)** and on CT-MR **(b, d, f, h)** (T1-weighted + Gd) for four different patients.

In general, a large variation in interobserver agreement was found for the delineated tumors on CT as well as for CT-MR delineations (Table 1, Figures 2 and 3).

The mean CIgen for CT was significantly larger (0.61, SD 0.12, range 0.29-0.77, 95% confidence interval: 0.56-0.67) compared to CT-MR (0.57, SD 0.15, range 0.17-0.80, 95% confidence interval: 0.50-0.64) (p = 0.032).

Although the smallest CIgens were observed for the smallest tumors (Table 1), no relation between CIgen and the mean GTV volume for CT as well as for CT-MR delineations was observed (CT: rho = 0.28 p = 0.24, CT-MR: rho *=* 0.31 p = 0.18).

### Clinical impact

When applying tight margins, for 12 of the 20 patients the largest GTV contour was not covered by all the PTVs. When using clinical margins this number decreased to two of the 20 patients. The anatomical sites where the GTV contour was not encompassed by the PTV contours were mostly in cranial and caudal direction.

## Discussion

The present study on supraglottic laryngeal carcinoma demonstrates that adding MR-images to CT resulted in a decrease in interobserver agreement compared to the interobserver agreement of the CT-only delineation-session. Furthermore, the median GTV volume was larger on CT-MR compared to CT although there was no relation found between the GTV volume and the CIgen. Subjectively, the observers reported an increased visibility of anatomical details on MRI.

According to other studies based on head-and-neck cancer where MRI was compared with CT, Ahmed et al. [6] demonstrated that the delineated GTV volume for base of tongue tumors on MRI was almost two times larger compared to CT. They also reported a superior subjective visualization and delineation of base of tongue tumors on the MRI-scans relative to CT. Several other studies concluded the same for tongue and floor of the mouth cancer [15,16] and nasopharyngeal carcinoma [9].

Although there is agreement on the capacity of MRI to increase visibility in head-and-neck oncology, there is no agreement on the value of MRI for determination of the GTV. Rasch et al. [10] showed better interobserver agreement with matched CT-MRI, for target delineation in nasopharynx cancer compared to CT alone. A large improving factor on the interobserver agreement was the decision to include entire anatomical structures invaded by tumor. A previous study done by Rasch et al. [7] reported that for six patients with advanced head-and-neck carcinoma, the delineated GTVs and interobserver agreement was better for delineations on MRI (with CT-images available) than on CT (with MR-images available). However, no difference between one single observers’ mean GTV volume delineated on CT and on MRI for oropharyngeal, laryngeal and hypopharyngeal tumors was found by Daisne et al. [11]. Additionally, a study performed by Geets et al. [8] showed no clinical advantage of MRI over CT in terms of volume determination and interobserver agreement for pharyngo-laryngeal tumors. Concerning the design of the study, this study [8] was the only one that, to some extent, resembled ours. However, we were not able to adequately compare our findings with results from the mentioned study because MRI was used without CT for delineating. Furthermore, the use of a different metric to quantify the interobserver agreement, based on area of overlap between contours, hampers a detailed comparison. In general, a wide variety of metrics is used to quantify the interobserver agreement in delineation studies for example: Dice similarity coefficient, common to encompassing volume ratio and Jaccard index [22,24,25].

Since the GTV is extended by margins to correct for several factors such as microscopic disease, movement and setup inaccuracies, the PTV is larger than the GTV. Our analysis indicates that large conventional margins partly compensate for the interobserver variation. However, when evidence-based tight margins are applied the interobserver variation for delineating the GTV might result in inadequate dose coverage of the GTV.

Tumor recurrence was diagnosed for two patients in this study. Due to the development in radiotherapy treatment schedules and tumor treatment planning between 2005 and 2009 we are not able to draw conclusions from this finding concerning treatment outcomes. Furthermore, the treatment plan was based on the delineation from the treating radiation-oncologist while the delineations in this study were used for research purposes.

In our study a dedicated MR protocol for radiotherapy GTV delineation was applied. This protocol has been used at our department since 2005. Care was taken to optimize the MR-image quality for radiotherapy purposes [17]. For the majority of the patients, MR-Image quality was considered “good”. However, the introduction of 3.0 Tesla and recently 7.0 Tesla MRI scanners and the development of new fast scan protocols might further optimize MR image quality.

A shortcoming of the studies of Daisne [11] and Geets [8] was the use of a multipurpose bodycoil as receiver coil for MR-imaging. Rasch [7] used a head coil for MR-imaging only without a mask or external markers, causing a decrease in image quality.

Although, in our study, the observers reported a subjectively increased visibility of anatomical details on MRI compared to CT, this did not improve agreement between observers. On the contrary, interobserver agreement was decreased. Apparently, additional MRI information resulted in more options to interpret the imaging data, resulting in a greater variation in delineations and an increase in delineated volumes. The inclusion of areas of doubt in the GTV, as described in our delineation guidelines, further increased these variations and volumes. In our opinion, the increased visibility of anatomical details on MRI might be of value in radiotherapy practice when it is clear how to combine the information of different MR-sequences when delineating the GTV. To maximize the benefits of high-precision radiation techniques, the gross tumor volume (GTV) should be determined as accurate as possible. Clear guidelines for interpretation and GTV delineation of laryngeal carcinoma could therefore be very useful. To develop these guidelines, a validation-study with total laryngectomy specimens is currently being performed at our institution. In that study, tumor tissue is identified based on pathological findings and compared with GTV delineations on different image modalities [26].

The large variation in interobserver agreement for the GTVs delineated on CT as well as for CT-MR delineations (Table 1) suggests that for some tumors it was more difficult to delineate the GTV compared to others. In some cases, this might have been influenced by a moderately decreased image quality. In our opinion, this variation was mostly caused by differences in location and characteristics of the tumor, and difficulties to distinguish tumor borders.

For the two T4 stage tumors, the differences between the delineated volumes on CT and CT-MR were the largest. This might be explained by the presence of edema that is increased in larger tumors and which could cause an increase in delineated volume on CT-MR since MR is superior in visualizing soft tissues (e.g. edema) [12-14]. The observers also included more cartilage in their GTV on CT-MR compared to CT only. Besides the capacity of MRI to increase visibility of soft tissue, MRI might have an improved visibility for cartilage invasion compared to CT. Research performed by Becker et al. supports this presumption [14,19]. Since there was no histopathological data available for this study we are not able to further investigate this finding.

The CT-images used in this study were obtained on 2 different CT-scanners and slice thickness varied between 2 and 3 mm. This did not influence the results since there were no remarkable differences between the CIgens comparing the two scanners. Besides, no difference in image quality and no specific matching related problems were reported.

## Conclusions

The interobserver agreement was decreased in the CT-MR session compared to the CT only delineations and mean delineated volume on CT-MR was larger compared to CT. At this point MR has no objective added value concerning the CIgen outcomes. The increased visualization of anatomical details on MRI might lead to an increased interobserver agreement and more accurate GTV estimation only when clear guidelines for interpretation and delineation of MR-images of laryngeal tumors are present.

## Additional files

Additional file 1:
**Delineations of three observers on CT for patient 2.** Delineations of three observers on contrast enhanced CT for patient number 2. From left to right: transversal, sagittal en coronal views. Additional file 2:
**Delineations of three observers on CT-MR for patient 2.** Delineations of three observers on for the CT-MR session for patient 2. Delineations were performed on the contrast enhanced CT with co-registered MR simultaneously visible (CT images are not shown). Upper left: transversal T1-weighed view. Lower left: transversal T1-weighted + gadolinium view. Upper right: coronal T1-weighted view. Lower right: sagittal T1-weighted view. Additional file 3:
**Delineations of three observers on CT for patient 11.** Delineations of three observers on contrast enhanced CT for patient number 11. From left to right: transversal, sagittal en coronal views. Additional file 4:
**Delineations of three observers on CT-MR for patient 11.** Delineations of three observers on for the CT-MR session for patient 11. Delineations were performed on the contrast enhanced CT with co-registered MR simultaneously visible (CT images are not shown). Upper left: transversal T1-weighed view. Lower left: transversal T1-weighted + gadolinium view. Upper right: coronal T1-weighted view. Lower right: sagittal T1-weighted view.
